# Close Interactions between Mesenchymal Stem Cells and Neuroblastoma Cell Lines Lead to Tumor Growth Inhibition

**DOI:** 10.1371/journal.pone.0048654

**Published:** 2012-10-31

**Authors:** Giovanna Bianchi, Fabio Morandi, Michele Cilli, Antonio Daga, Chiara Bocelli-Tyndall, Claudio Gambini, Vito Pistoia, Lizzia Raffaghello

**Affiliations:** 1 Laboratory of Oncology, Istituto Giannina Gaslini, Genoa, Italy; 2 Technology Transfer, Animal Research Facility, San Martino-National Institute for Cancer Research, Genoa, Italy; 3 Department of Translational Oncology, National Institute for Cancer Research, Genoa, Italy; 4 Department of Rheumatology, University of Basel, Switzerland; 5 Laboratory of Pathology, Istituto Giannina Gaslini, Genoa, Italy; The University of Chicago, United States of America

## Abstract

Mesenchymal stem cells (MSCs) have attracted much interest in oncology since they exhibit marked tropism for the tumor microenvironment and support or suppress malignant cell growth depending on the tumor model tested. The aim of this study was to investigate the role of MSCs in the control of the growth of neuroblastoma (NB), which is the second most common solid tumor in children. *In vivo* experiments showed that systemically administered MSCs, under our experimental conditions, did not home to tumor sites and did not affect tumor growth or survival. However, MSCs injected intratumorally in an established subcutaneous NB model reduced tumor growth through inhibition of proliferation and induction of apoptosis of NB cells and prolonged the survival of hMSC-treated mice. The need for contact between MSCs and NB cells was further supported by *in vitro* experiments. In particular, MSCs were found to be attracted by NB cells, and to affect NB cell proliferation with different results depending on the cell line tested. Moreover, NB cells, after pre-incubation with hMSCs, acquired a more invasive behavior towards CXCL12 and the bone marrow, i.e., the primary site of NB metastases. In conclusion, this study demonstrates that functional cross-talk between MSCs and NB cell lines used in our experiments can occur only within short range interaction. Thus, this report does not support the clinical use of MSCs as vehicles for selective delivery of antitumor drugs at the NB site unless chemotherapy and/or radiotherapy create suitable local conditions for MSCs recruitment.

## Introduction

Human mesenchymal stem cells (hMSCs) are a heterogeneous population of non-hematopoietic multipotent cells endowed with self renewal ability that can differentiate into cells of mesodermal lineage [1], [2]. Although hMSCs may be isolated from many adult tissues, the bone marrow is the main organ from which hMSCs are derived and characterized [3]. There is a general consensus that hMSCs i) are plastic-adherent cells under standard culture conditions, ii) express variable levels of CD105, CD73, CD44, CD90, CD71, CD217, disialoganglioside GD_2_, and the stromal marker STRO-1, and lack expression of hematopoietic markers such as CD45, CD34 and CD14 or the co-stimulatory molecule CD80, and iii) differentiate into osteoblasts, condrocytes and adipocytes [4], [5].

hMSCs have a crucial role in the formation of the hematopoietic stem cell (HSCs) *niches* in the bone marrow by shielding HSCs from apoptotic and differentiation signals and supporting maintenance and self-renewal of HSCs [5], [6].

In addition, hMSCs exert immunoregulatory activities by suppressing T- and B-cell lymphocyte proliferation, dampening the generation of mature myeloid dendritic cells and inhibiting proliferation, cytokine production and cytotoxic activity of resting natural killer (NK) cells [7]–[11].

Interestingly, hMSCs exhibit an innate tropism for injured tissues as well as for tumor microenvironment due to attraction by similar factors secreted by wounds and tumors [12]. Different studies demonstrated the capacity of hMSCs to home to tumors and participate in tumor stroma formation [13], [14]. The tropism of hMSCs for tumors has been exploited by using MSCs as gene-delivery vehicles for anticancer agents, and this approach has proven to be successful in different preclinical models of cancer [15]–[21]. Although some studies showed that hMSCs may inhibit tumor growth [22]–[26], others have demonstrated opposite effects [27]–[36]. hMSCs may favor tumor growth through different mechanisms such as promotion of the metastatic potential and neoangiogenesis, preservation of the self-renewal ability of cancer cells, and prevention of tumor cell recognition by the immune system [27]–[31], [37]. Conversely, hMSCs may exert antineoplastic activities by downregulating proliferative capacity of tumor cells and inducing expression of apoptotic molecules in the latter cells [22], [26], [36].

To the best of our knowledge, scanty information is available about the effects of MSCs on the growth of neuroblastoma (NB), the second most common solid tumor in children that presents in a half of the patients with metastatic disease at diagnosis involving mainly bone marrow, bone, lymph nodes and liver [38].

Here we have investigated the functional interactions between MSCs and NB cells in *in vitro* and *in vivo* models and the impact of MSCs on tumor growth. We found that hMSCs inoculated intravenously (i.v.) in different NB models did not localize to the tumor mass and did not affect NB tumor growth. In contrast, in a subcutaneous localized NB model, hMSCs administered intratumorally exerted an antineoplastic effect through significant inhibition of cell proliferation and induction of apoptosis. *In vitro* studies highlighted the need for close cell-to-cell interaction in order to detect functional changes in MSCs incubated with NB cells and *viceversa*.

Our finding seem not to support the use of MSCs as vehicles for selective delivery of antitumor drugs at NB tumor site, however we cannot exclude that these results may be related to specific and unique features of the NB cell lines used.

## Results

### *In vivo* effects of mesenchymal stem cells in pseudo-metastatic neuroblastoma models

In order to evaluate the effects of hMSCs on *in vivo* NB growth and progression, we developed a pseudometastatic NB model by i.v. injection of human NB Htla-230, which represents a NB cell line able to induce metastases upon systemic inoculum.

**Figure 1:**
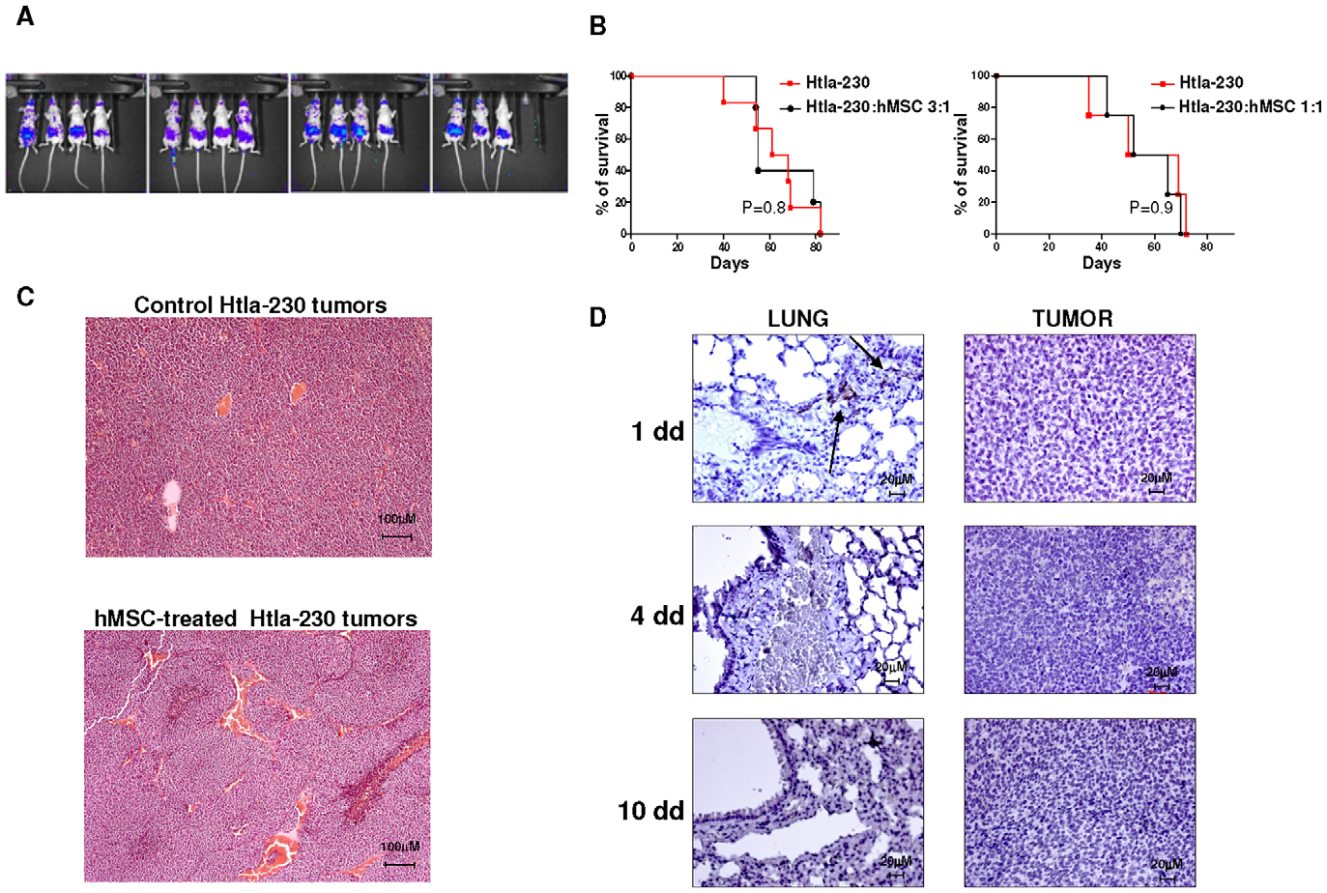
Survival curves and immunohistochemical analysis of human Htla-230 neuroblastoma bearing mice treated with human mesenchymal stem cells. Panel A. Athymic mice (Nude-nu) were i.v. injected with ffLUC-Htla-230 (3×10^6^ cell/mouse; n = 15). Tumor establishment occurred 14 days after tumor cell inoculum as assessed by bioluminescence imaging. Panel B. Athymic mice (Nude-nu) were injected in the tail vein with Htla-230 (3×10^6^ cell/mouse) and treated i.v. with hMSCs (1×10^6^ cells/mouse, n = 6 or 3×10^6^ cells/mouse, n = 5) or saline solution (n = 6) 14 days after tumor cell inoculum. Survival curves were constructed by using the Kaplan–Meier method. Statistical analysis of different treatment groups was performed by Peto's log-rank test. Panel C. Representative hematoxylin-eosin-staining of tumors surgically removed from control and hMSC-treated Htla-230-bearing mice is shown. Original magnification 10x. Panel D. Representative immunohistochemical CD90 staining of lungs and tumors surgically removed from hMSC-treated Htla-230 bearing mice at different times after hMSC i.v. inoculum. Arrows indicate hMSCs positive for CD90. Original magnification 40x.

First, we demonstrated that Htla-230 cells transfected with firefly luciferase (ffLUC) (ffLUC-Htla-230) (3×10^6^ cells/mouse) and i.v. injected in athymic nude mice induced the formation of an abdominal tumor mass which started to become established 14 days after tumor cell inoculum, as assessed by bioluminescence (Fig. 1 Panel A).

Next, hMSCs (1×10^6^ cells/mouse with NB:hMSC ratio of 3∶1) were injected i.v. 14 days after Htla-230 NB cell inoculum. The NB:hMSC ratio of 3∶1 has been chosen since it is in the range of what is commonly described by different studies [37]. Survival time was used as the end point to determine the effects of hMSCs treatment on tumor growth. At this NB:hMSC cell ratio, hMSCs did not exert any effect on metastatic tumor growth at any follow-up time (data not shown) nor did they affect the survival of NB Htla-230 bearing mice (P = 0.8 by Log Rank test) (Fig. 1 Panel B). Of note, increasing the amount of injected hMSCs to a NB:hMSC ratio of 1∶1 did not affect the metastatic tumor growth as well as the survival of NB Htla-230 bearing mice (Fig. 1 Panel B).

An histological analysis of control and hMSC-treated Htla-230 tumors showed similar features including the presence of small undifferentiated tumor cells with high cellularity, a mitosis-karyorrhexis index >4%, numerous vascular septa, high mitotic index and some necrotic foci (Fig. 1 Panel C). In addition, tumor cells in both the experimental groups exhibit an high nucleus:cytosol ratio and are positive for typical NB markers such as NB84, CD56 and N-CAM (data not shown). All these histological features are consistent with a diagnosis of schwannian stroma poor NB in both control and hMSC-treated mice.

In order to detect migrating hMSCs in NB bearing mice, we performed an immunohistochemical analysis of Htla-230 NB bearing mice at different times after hMSC i.v. inoculum. To this end, serial tissue sections of lungs and tumors were stained with CD90 mAb which detects hMSCs. As shown in Figure 1 Panel D, 1 day after hMSC inoculum, CD90^+^ hMSCs were detected in the lungs of Htla-230 bearing mice with perivascular localization, and disappeared at day 4 and day 10 after MSC inoculum. In contrast, CD90^+^ hMSCs were never detected in the tumor at any time after hMSCs i.v. inoculum. The same immunohistochemical analysis, perfomed on the lungs and tumors of Htla-230 NB bearing mice not injected with hMSCs, revealed complete lack of CD90^+^ cells in any organ tested and at any time (data not shown).

In a set of experiments, we also tested the effects of hMSCs in an orthotopic NB model by inoculation of human NB SH-SY5Y cells (1×10^6^cell/mouse) in the fat pad of the adrenal gland of athymic nude mice. This model has been selected since it mimics more closely the situation observed in NB patients with disseminated disease. hMSCs did not exert any effect on primary and metastatic tumor growth (data not shown) and nor did they affect the survival of NB bearing mice (P = 0.17 by Log Rank test) (Fig. S1).

These results suggested that hMSCs were not recruited to NB microenvironment, under our experimental conditions, after i.v. injection, or, alternatively, localized to the tumor site but did not exert any functional effect on NB cells.

### Mesenchymal stem cell biodistribution in neuroblastoma experimental models

Since antitumor immune responses may generate inflammatory signals contributing to MSC recruitment to the tumor site, we investigated whether MSCs affected tumor growth in NB bearing immunocompetent mice.

In order to detect MSCs in NB tumor bearing mice, murine MSCs were stably transfected with firefly luciferase (ffLuc-mMSCs) and i.v. injected in murine NB bearing mice as well as in healthy controls at different times after tumor cell inoculum.

**Figure 2:**
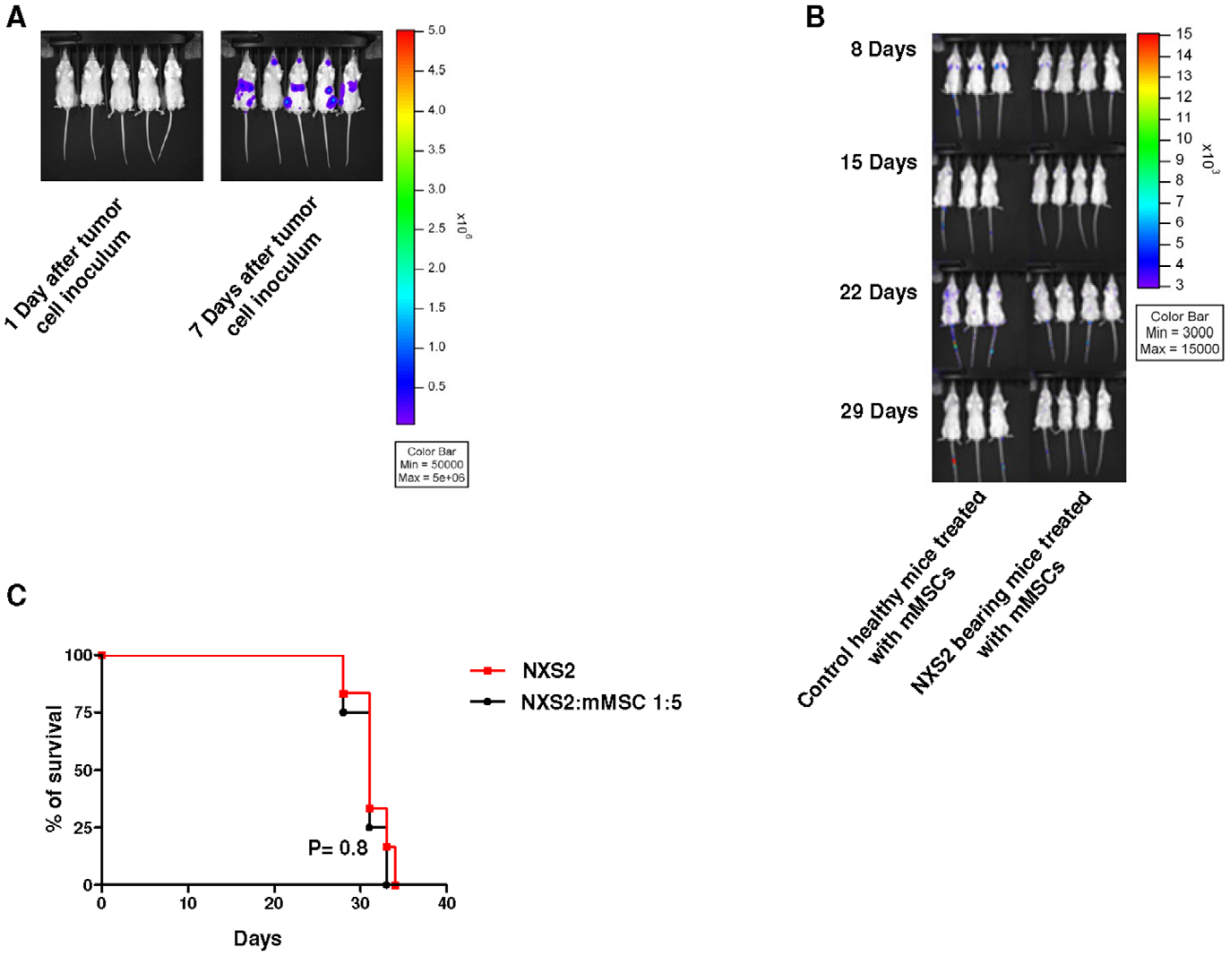
Survival curves and murine mesenchymal stem cell distribution within neuroblastoma bearing immunocompetent mice. Panel A. A/J mice were injected in the tail vein with ffLUC-NXS2 cells (2×10^5^ cells/mouse; n = 5) and imaged by bioluminescence imaging 1 and 7 days after tumor cell inoculum. Panel B. A/J mice were i.v. injected or not with NXS2 cells (2×10^5^ cells/mouse) and treated with ffLUC-mMSC (1×10^6^ cells/mouse) 7 days after tumor cell inoculum. Control group included 3 healthy mice treated with mMSCs whereas NXS2 bearing mice group includes 4 mice injetced with NXS2 cells and treated with mMSCs. The animals were imaged by bioluminescence 8, 15, 22, 29 days after tumor cell inoculum. This is a representative experiment out of three performed. Panel C. A/J mice were inoculated in the tail vein with NXS2 cells (2×10^5^ cells/mouse) and treated with syngeneic ffLUC-mMSC (1×10^6^ cells/mouse) or saline seven days after tumor cell inoculum. Control group included 6 mice only injected with tumor cells, whereas mMSC-treated groups included 6 mice. Survival curves were constructed by using the Kaplan–Meier method. Statistical analysis of different treatment groups was performed by Peto's log-rank test. This is a representative experiment out of three performed.

In a pilot experiment we demonstrated that immunocompetent A/J mice inoculated i.v. with the syngeneic murine NB ffLUC-NXS2 cell line (2×10^5^ cells/mouse) exhibited established masses starting from seven days after tumor cell inoculums, as assessed by bioluminescence (Fig. 2 Panel A).

Next, immunocompetent A/J mice were inoculated or not with i.v. with the syngeneic murine NB NXS2 cell line (2×10^5^ cells/mouse) and treated with intravenous injection of syngeneic ffLuc-mMSCs (1×10^6^ cells/mouse) 7 days after tumor cell inoculum. As shown in Figure 2 Panel B, only few mMSCs were detected by bioluminescence in the lungs of control healthy animals and NXS2-bearing mice days following tumor cell inoculum and were no longer visualized 15, 22 or 29 days. NB bearing mice treated with mMSCs 7 days after NB cell inoculum showed a survival comparable to that of untreated NB bearing mice (Fig. 2 Panel C) (Ctr *vs* mMSC P = 0.9, by Log Rank Test).

Similar results were obtained when control or NB bearing mice received syngeneic ffLuc-mMSCs (1×10^6^ cells/mouse) 1 day after tumor cell inoculum when tumor metastasis were not established (not shown).

Similarly, hMSCs were transfected with firefly luciferase (ffLuc-hMSCs) and i.v. injected in Htla-230 NB bearing nude mice. FfLuc-hMSCs were not detectable at any time after cell injection and no differences in term of long term survival were found between hMSCs-treated and control mice (data not shown).

### *In vitro* functional interactions between mesenchymal stem cells and neuroblastoma cells

**Figure 3:**
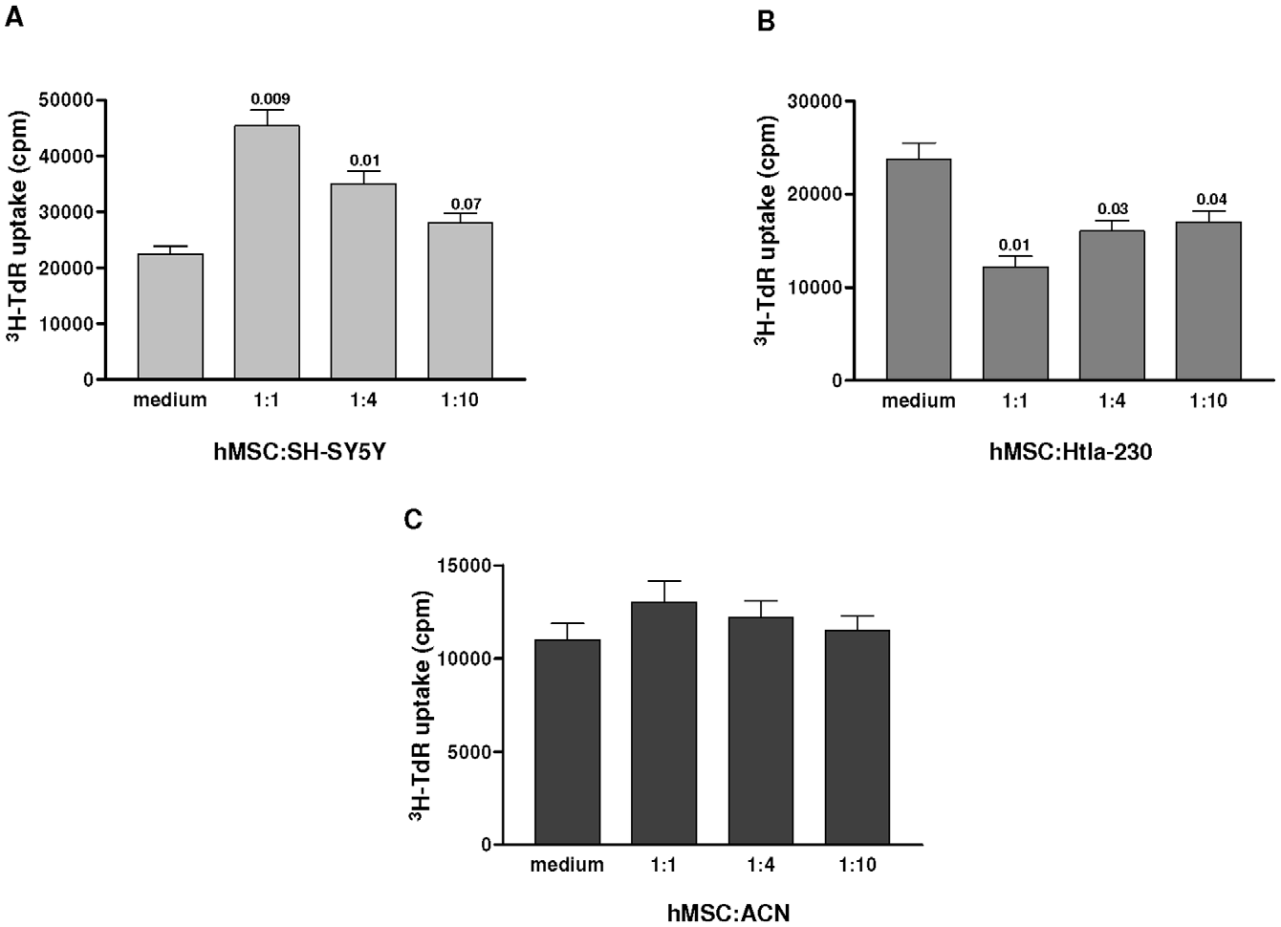
Effects of human mesenchymal stem cells on neuroblastoma cell proliferation. SH-SY5Y (Panel A) and Htla-230 (Panel B) and ACN (Panel C) NB cells were cultured in 96-well plates for 3 days in presence or absence of hMSCs at different ratio. After 3 days of co-culture, the cells were pulsed with ^3^H-thymidine for 18 hours and analyzed. The data are expressed as mean value ± SD from three different experiments. Statistical analysis was performed by Student's t test with Welch's correction.

In order to investigate the reciprocal interactions between MSCs and NB cells, we tested whether hMSCs could modulate the survival/growth or migration of NB cells in an *in vitro* co-culture system. Three human NB cell lines including SH-SY5Y, Htla-230, and ACN cells, employed for our *in vivo* experiments, were cultured in presence or absence of different concentrations of irradiated hMSCs and tested for proliferation after 4 days. As shown in Figure 3 Panels A and B, the effects of hMSCs on NB cell proliferation were variable. Thus, hMSCs significantly increased proliferation of SH-SY5Y (Fig. 3 Panel A) and reduced that of Htla-230 cells (Fig. 3B) in a dose dependent manner, whereas proliferation of ACN cells was not affected (Fig. 3 Panel C).

**Figure 4:**
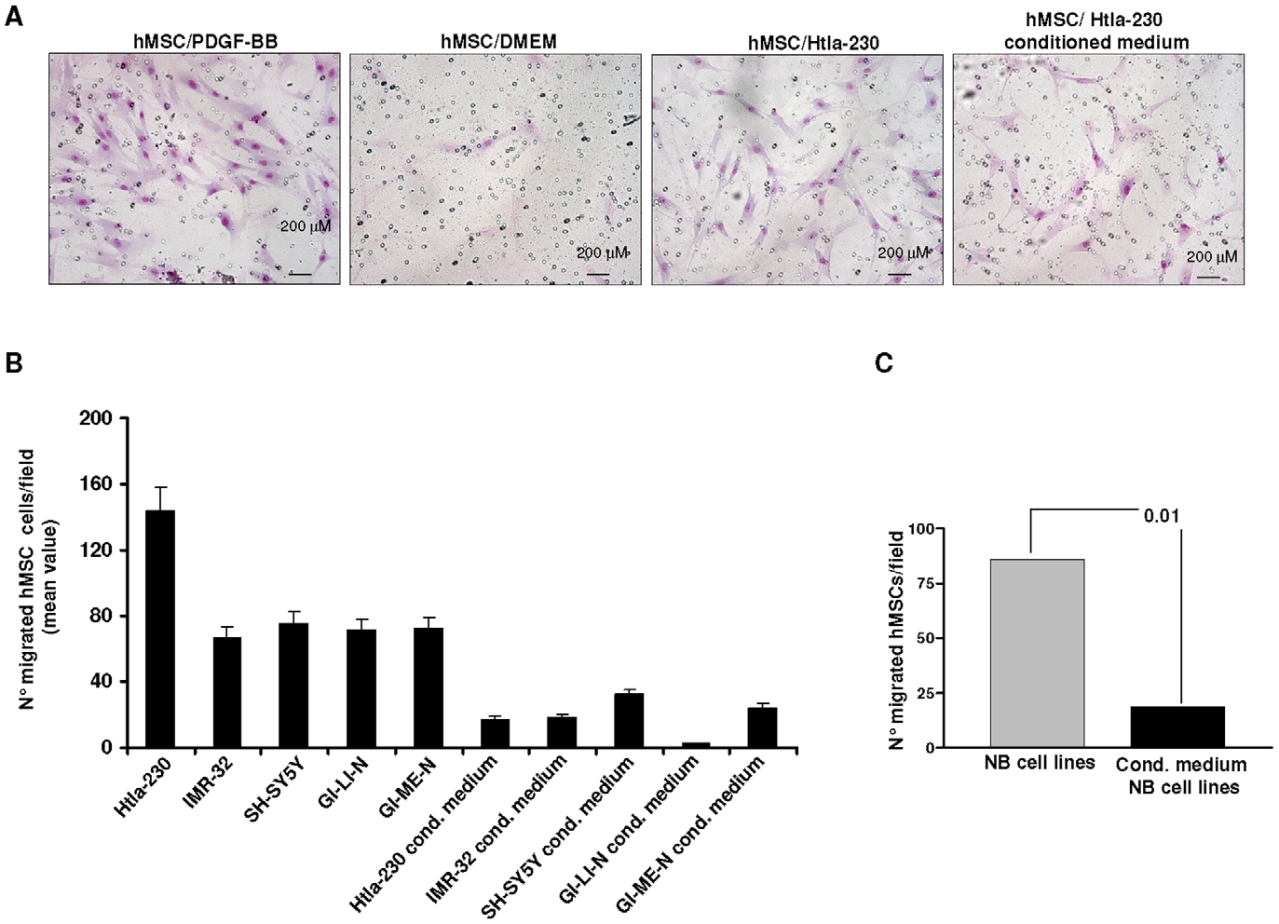
Human neuroblastoma cells attract human mesenchymal stem cells *in vitro*. Human MSCs, placed in the upper wells of transwell system, were separated by an 8 μm membrane from NB cells or NB cell conditioned medium that were dispensed in the lower well. Negative and positive controls were represented by serum-free medium (DMEM) and PDGF-BB at 100 ng/ml, respectively. Migration was quantified by counting migrated cells under the microscope after cell staining with May Grunwald. Panel A shows representative images of migrated hMSCs to DMEM, PDGF-BB, Htla-230 NB cells, Htla-230 conditioned medium. Original magnification 100x. Panel B shows quantification of migrated hMSC to different NB cells and NB conditioned medium. The data represent mean values obtained from the count of 10 fields/well. Bars represent SD. Panel C shows the results obtained by pooling all the data obtained from hMSC migration to NB cell lines and NB cell conditioned medium. Statistical analysis was performed by Unpaired t test with Welch's correction.

Next, we evaluated the migration of hMSCs toward NB cells using Transwell plates. hMSCs, placed in the upper wells of Transwell, were separated by an 8 μm membrane from NB cells or NB cell conditioned medium, that were dispensed in the lower well. Negative and positive controls were represented by serum-free medium (DMEM) and PDGF-BB (100 ng/mL), respectively. Figure 4 Panels A–C shows that hMSCs were significantly attracted by different NB cell lines and, to a lesser extent, by supernatants from the latter cells. After pooling together the results obtained from hMSCs *vs* NB cell lines and hMSCs *vs* supernatants thereof, hMSCs were found to be more significantly attracted by NB cells than by their supernatants (Fig. 4 Panel C: P = 0.01, Unpaired t test with Welch's correction).

In a further set of experiments, we investigated whether different NB cells (SH-SY5Y, GI-LI-N and Htla-230), chosen for their ability to *in vivo* metastasize upon systemic or orthotopically administration and for their different constitutive CXCR4 expression, acquired higher migration capability to CXCL12, after preincubation with hMSCs. CXCL12 was selected for these experiments since it was been found to promote NB cell invasiveness in different pre-clinical NB models [39], [40]. According to other studies [41], SH-SY5Y, GI-LIN and Htla-230 NB cells expressed variable constitutive levels of CXCR4 (SH-SY5Y mean %±SD: 3.5±0.3; GI-LI-N mean %±SD: 11±0.1; Htla-230 mean %±SD: 15±0.1).

**Figure 5:**
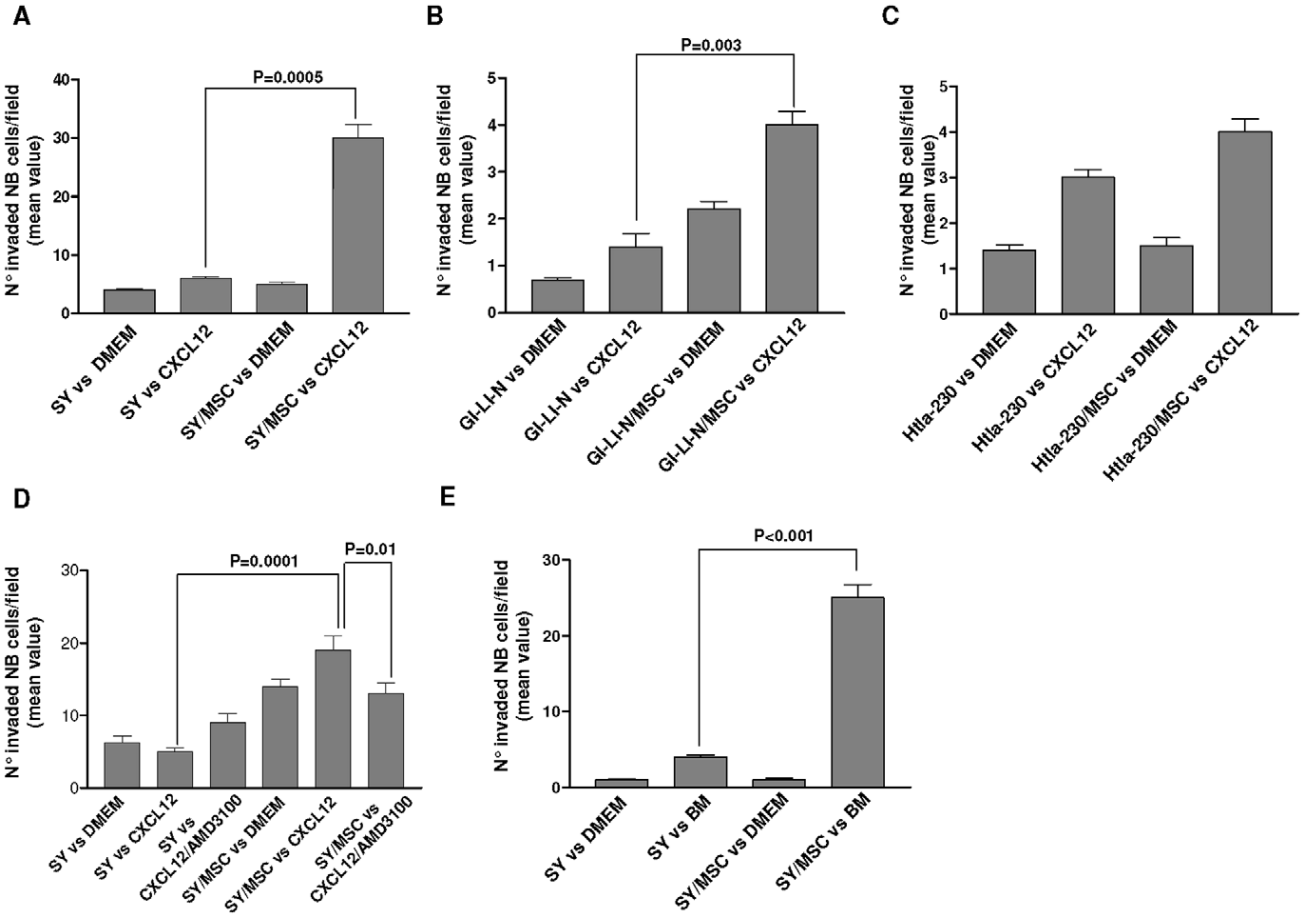
Invasiveness of neuroblastoma cells after contact with human mesenchymal stem cells. SH-SY5Y, Htla-230 and GI-LI-N NB cells incubated or not with hMSCs in a transwell system for 24 hours, were placed in the upper wells on Matrigel®-coated invasion chambers and separated by an 8 μm membrane from CXCL12 or bone marrow sample. After 24 hours, NB cells migrated into the lower chamber were stained and counted after May Grunwald-Giemsa. Panels A–C show the invasiveness of SH-SY5Y (Panel A), GI-LI-N (Panel B) and Htla-230 (Panel C) to CXCL12. Panels D shows the invasiveness of SH-SY5Y to CXCL12 in presence or absence of CXCR4 antagonist AMD3100 that was added to some wells. Panel E shows the invasiveness of SH-SY5Y to the bone marrow of an healthy donor. The data are expressed as mean values obtained from the count of 10 fields/well. Bars are SD. Statistical analysis was performed by Unpaired t test with Welch's correction.

Similarly to the experiments on cell proliferation, the effects of hMSCs on NB cell migration to CXCL12 were variable. As shown in Figure 5, SH-SY5Y (Panel A) and GI-LI-N (panel B) NB cells pre-incubated with hMSCs in a transwell system for 24 hours, displayed an enhanced migratory capability to CXCL12, in comparison to the same cells pre-incubated with medium alone (DMEM) (SY to CXCL12 *vs* SY/hMSCs to CXCL12 P = 0.0005; GI-LI-N to CXCL12 *vs* GI-LI-N/hMSCs to CXCL12 P = 0.003; Unpaired t test with Welch's correction). In contrast, Htla-230, pre-incubated with hMSCs did not migrate to CXCL12 at significantly higher extent in comparison to the same cells pre-incubated with medium alone (Fig. 5 Panel C). According to the constitutive expression of CXCR4, the differences in migratory potential of SH-SY5Y, GI-LI-N and Htla-230 in response to CXCL12 seem not to be related to their relative CXCR4 expression.

In order to further clarify the role of CXCL12/CXCR4 axis in NB migration, we tested the migratory capability of SH-SY5Y NB cells pre-incubated with hMSCs for 24 hours in presence or absence of the CXCR4 inhibitor AMD3100. Figure 5 Panel D shows that NB cells pre-incubated with hMSCs displayed enhanced migratory capability to CXCL12 (SY to CXCL12 *vs* SY/hMSCs to CXCL12 P = 0.0001; Unpaired t test with Welch's correction). Such effect was abrogated by AMD3100 (SY/hMSCs to CXCL12 *vs* SY/hMSCs to CXCL12/AMD3100 P = 0.01).

In a set of experiments, we also investigated whether SH-SY5Y cells, after preincubation with hMSCs acquired higher invasion capability to the bone marrow i.e. the primary site of neuroblastoma metastases. As shown in Figure 5 Panel E, SH-SY5Y NB cells, pre-incubated with hMSCs Matrigel®-coated invasion chambers for 24 hours, displayed an enhanced invasive potential to the bone marrow, in comparison to the same cells pre-incubated with medium alone (DMEM) (SY to bone marrow *vs* SY/hMSCs to bone marrow P<0.0001; Unpaired t test with Welch's correction).

These results suggest that hMSCs exhibited similar migratory capabilities toward different NB cell lines, but exerted variable effects on NB cell proliferation and migration depending on the cell line tested.

### Mesenchymal stem cell inhibition of *in vivo* neuroblastoma growth in a subcutaneous tumor model

In order to investigate *in vivo* close interactions between MSCs and NB cells mimicking those described above in an *in vitro* model, we set up a localized NB model by implantation of human NB ACN cells (3×10^6^ cells/mouse) subcutaneously (s.c.) into the right flank of athymic nude mice. This cell line was selected for such experiments since it was the only one, among those tested in this study, that proved to be tumorigenic following s.c. inoculum. When the s.c. tumors reached the size of 100 mm^3^, hMSCs (1×10^6^ cells/mouse) were intratumorally injected.

**Figure 6:**
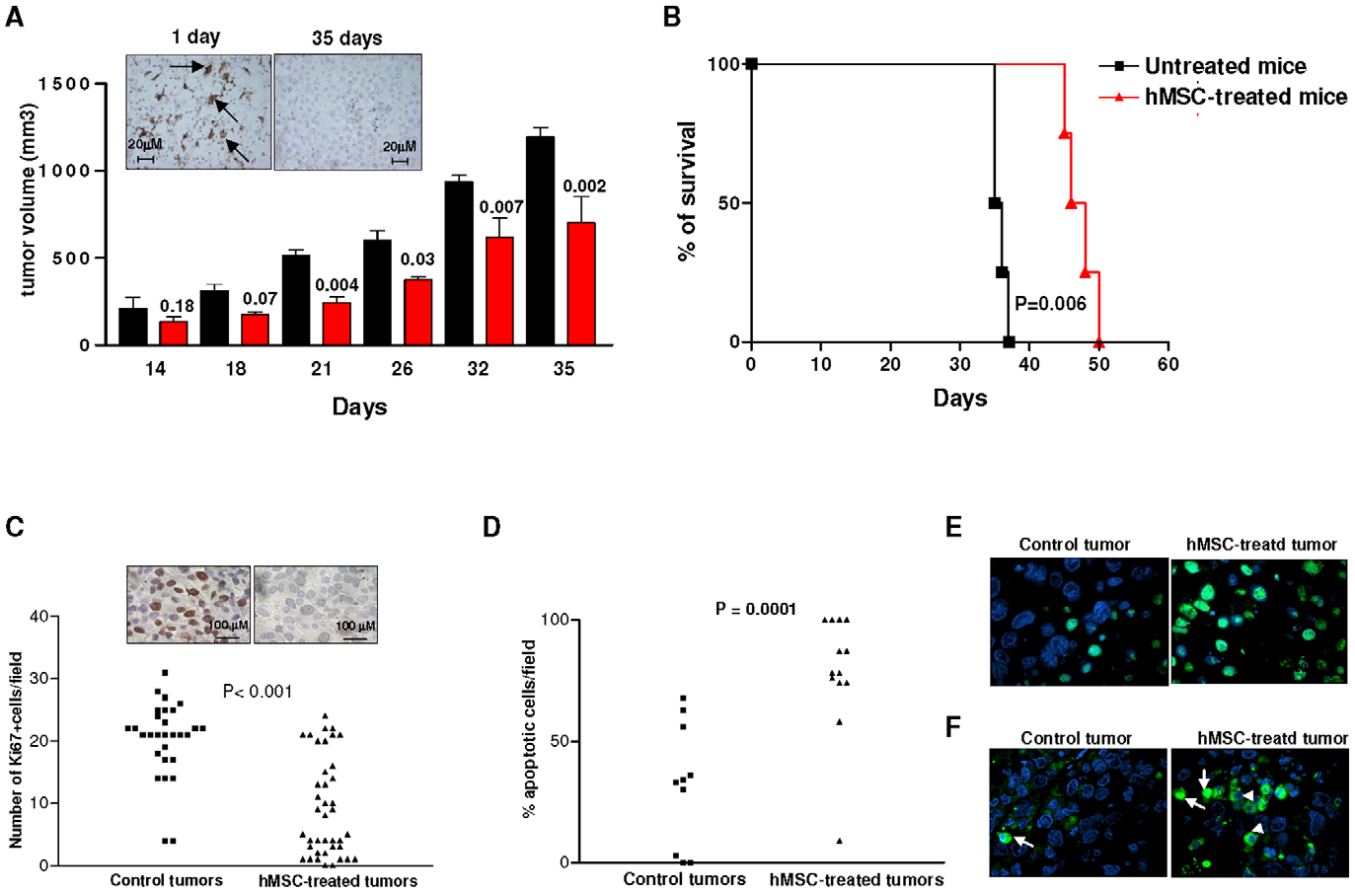
Effects of human mesenchymal stem cells after intratumoral injection in neuroblastoma tumors. Panel A. Athymic mice were injected with human NB ACN cells (3×10^6^ cells/mouse) subcutaneously (s.c.) into the right flank. When the tumor size reached 100 mm^3^, hMSCs (1×10^6^ cells/mouse, 5 mice, red bars) or saline solution (control group: 5 mice, black bars) were injected intratumorally. Tumor volume was measured at different times and p values were calculated using Student's t test with Welch's correction. A representative experiment out of two performed is shown. Inset panel A shows a representative CD90 immunohistochemical staining of hMSC-treated ACN tumors surgically removed 1 day or 35 days after hMSC inoculum. Arrows indicate MSCs positive for CD90. Original magnification 40x. Panel B. Survival curves of ACN-bearing mice untreated or treated with hMScs were constructed by using the Kaplan–Meier method. Statistical analysis of different treatment groups was performed by Peto's log-rank test. This is a representative experiment out of two performed. Panel C. Paraffin-embedded tumor sections from control and hMSC-treated mice were stained for the cell proliferation marker Ki67. Data are expressed as mean value of Ki67 positive cells counted in 10 fields/slide. Inset panel B shows a representative staining of control and hMSC-treated ACN tumors with Ki67. Original magnification 100x. P values were calculated using Unpaired t test with Welch's correction. Panel D. Paraffin-embedded tumor sections from control and hMSC-treated mice were stained for detection of apoptosis with terminal deoxynucleotidyl transferase-mediated dUTP nick end labeling [TUNEL] assay. Data are expressed as mean value of TUNEL positive cells counted in 10 fields/slide. p values were calculated using Unpaired t test with Welch's correction. Panel E. Representative staining of control and hMSC-treated ACN tumors with TUNEL. Green cells are TUNEL positive. Panel F. Representative immunofluorescent staining of control and hMSC-treated ACN tumors with anti-caspase-3 mAb. Arrow indicate positive cells with nuclear activated caspase-3 localization; arrowheads indicate positive cells with cytosolic activated caspase-3 localization.

Figure 6 Panel A shows that hMSCs inhibited significantly tumor growth, as assessed by measuring the tumor volume at different times after hMSC administration. CD90 positive hMSCs were detected in the tumor mass one day after hMSC injection, but were no longer detectable 35 days after hMSCs inoculum (Inset Panel Fig. 6 Panel A). According to the growth inhibitory effect mediated by hMSCs on ACN-tumors, hMSC-treated mice survived longer in comparison to untreated animals bearing ACN NB (P = 0.006 by Log Rank test) (Fig. 6 Panel B).

In order to evaluate the impact of hMSCs on tumor cell proliferation, viability, apoptosis and angiogenesis *in vivo*, we carried out immunohistochemical analyses on paraffin-embedded tissue sections of tumors from hMSC-treated as well as control mice sacrificed 18 days after treatment. As shown in Figure 6 Panel C, hMSC-treated tumors contained a statistically significant lower proportion of Ki67 positive proliferating cells compared to control tumors (p<0.0001; Unpaired t test with Welch's correction). A representative staining of hMSC-treated and control tumors with Ki67 mAb is shown in the inset panel of Figure 6 Panel C. Double staining of paraffin-embedded sections from control tumor and hMSC-treated tumors by TUNEL and DAPI demonstrated that hMSC-treated tumors contained significantly a higher percentage of apoptotic cells than control tumors (P<0.0001 Student's t test with Welch's correction) (Fig. 6 Panel D). A representative staining of hMSC-treated and control tumors is shown in Figure 6 Panel E. To identify the mechanism by which tumor cells undergo apoptosis after hMSC treatment, we evaluated the expression of activated caspase-3, that represents a key mediator of apoptosis of mammalian cells [42]. As shown in Figure 6 Panel F, immunofluorescence analysis of hMSC-treated ACN tumors revealed strong caspase-3 expression at the nuclear as well as the cytosolic level. In contrast, control tumors exhibited faint expression of activated caspase-3, mainly localized in the cytosol (Fig. 6 Panel F).

Finally, angiogenesis was not affected by hMSC treatment since the proportion of cells expressing the endothelial cell marker CD31 was superimposable in hMSC-treated and control tumors (not shown).

## Discussion

Different studies showed that MSCs promote tumor progression and metastasis [27]–[35], [43], while other studies reported that MSCs suppress tumor growth [22]–[26]. These discrepancies are likely attributable to one or more of the following variables, i) different tumor models, ii) heterogeneity of MSCs, iii) dose or timing of MSCs injected, and iv) animal host [37]. On the other hand, the ability of MSCs to localize to tumors make them attractive candidates for targeted delivery of anti-neoplastic drugs at the tumor site [15]–[21].

In this study we report the effects of MSCs on neuroblastoma, a neural-crest derived pediatric malignancy, often characterized by the presence of metastases in different organs such as bone marrow, bone, lymph nodes and liver. Although no information is available as to the effects of MSCs on NB growth, MSCs have been reported to play a critical role in osteoclast activation and consequent bone destruction in NB [44]. In particular, human NB cells, that form osteolytic lesions *in vivo*, do not produce *per se* osteoclast-activating factors but rather stimulate osteoclast activity in the presence of human bone marrow MSCs [44]. This alternative pathway of osteoclast activation involves non adhesive interactions between NB cells and bone marrow-derived MSCs, culminating in increased levels of MSC-derived interleukin-6, which is ultimately responsible for osteoclast activation [44].

Another study reported on the MSCs capability to differentiate into Schwann cells following inoculation in NB tumors, thus contributing to the formation of NB stroma [45].

Finally, tumor stromal cell cultures from pediatric tumors and MSCs derived from bone marrow aspirates of NB patients were found to share phenotypic and functional features, including inhibition of peripheral blood mononuclear cell proliferation and NK cell-mediated cytotoxicity [46].

A recent clinical study demonstrated the safety and the efficacy of infusing autologous MSCs infected with ICOVIR-5, a new oncolytic adenovirus, for treating metastatic NB patients. Tolerance to treatment was excellent and a complete clinical response was documented in one case [47].

Our experiments performed with human NB cell lines injected in immunodeficient mice showed that hMSCs did not exert any effect in a pseudometastatic NB model that mimics human NB growth and dissemination. On the basis of these results, we hypothesized that: i) MSCs did not migrate to NB tumors, or alternatively ii) MSCs migrated to NB tumors but did not exert any functional effect on NB cells.

MSCs delivered intravenously or intra-arterially have been shown to engraft within tumor microenvironment [12]. This tropism is likely due to an inflammatory milieu produced by tumors that is very similar to that produced by healing wounds [14]. Recently, damage-associated molecular patterns (DAMPs) and especially the DNA-binding molecule high-mobility group box 1 (HMGB-1) [48] have attracted interest. Upon release by necrotic tumor cells, DAMPs recruit and stimulate local proliferation of MSCs that differentiate into tumor-associated fibroblasts promoting tumor growth and angiogenesis [33], [49]. Strategies aimed at increasing the expression of inflammatory mediators in the tumor microenvironment or to cause tumor cell death, such as chemotherapy and/or radiotherapy, have been shown to significantly increase the recruitment of circulating MSCs to the tumor milieu [21].

Here we show that MSCs injected in the tail vein of NB bearing immunocompetent (NXS2 bearing mice) or immunodeficient (Htla-230 bearing mice) mice did not localize to the tumor site. Failure of MSCs to home to the NB microenvironment, under our experimental conditions, may be related to the fact that NB cells do not secrete sufficient chemoattractants for long range recruitment of MSCs. An additional contributory factor may be the paucity of inflammatory cells in NB stroma. In line with this observation, it has been shown in different experimental models that MSCs may have little propensity to localize to injured tissues although exerting paracrine biological effects [50], [51]. However, based to the fact that MSCs show different effects on different NB cell lines, we cannot also exclude that the lack of MSC recruitment in NB is likely a cell line specific phenomenon.

Although MSCs did not seed in NB microenvironment and did not affect the growth of pseudometastatic and orthotopic NB, the results obtained from an *in vitro* co-culture system indicated that MSCs were attracted by NB cells, and affected NB cell proliferation with different effects depending on the cell line tested. The variable effects of MSCs on NB cell line proliferation likely reflected intrinsic differences in the growth characteristics of these cell lines. Notably, NB cell supernatants were less efficient than NB cells themselves at attracting MSCs *in vitro*, indicating that a cross-talk between these cell types was required in order to stimulate optimal migration of MSCs.

In addition, we demonstrated that NB cells, upon *in vitro* incubation with MSCs, acquired a more invasive behavior towards the bone marrow, i.e. the primary site of neuroblastoma metastases, and to CXCL12 which is highly expressed by bone marrow and NB cells [40], [52]. Interestingly, we found that AMD3100, a synthetic CXCL12 antagonist known to block CXCR4 function [53], abrogated the enhanced invasiveness of NB cells pre-incubated with MSCs, suggesting that the CXCR4/CXCL12 axis played a pivotal role in the MSC-mediated migration of NB cells. Accordingly, a recent study showed that MSCs are important for NB metastasis via the secretion of CXCL12 and that such effect can be inhibited by AMD3100 [54]. CXCR4/CXCL12 plays a relevant role in mediating interactions between NB cells and MSCs, however other molecules such as CXCR7 are involved in the migration of NB towards MSCs [54]. Of note, urokinase plasminogen activator and urokinase plasminogen activator receptor have been shown to mediate human MSC tropism to malignant solid tumors including NB [55].

Altogether, the discrepancies between the *in vivo* and *in vitro* findings discussed above may be explained by the fact that MSCs need to establish short range interactions with NB cells in order to exert any functional effect on the latter cells.

The final proof of this concept came from the results obtained in an *in vivo* subcutaneous model in which MSCs were injected into already established tumor nodules formed by the ACN NB cell line. In this model, MSCs inhibited significantly tumor growth by decreasing proliferation and inducing apoptosis of NB cells through activation of caspase-3.

Surprisingly, in our experimental conditions, MSCs did not exert any effect on tumor angiogenesis. In this respect, MSCs have been reported to promote tumor angiogenesis by secreting pro-angiogenic factors and by differentiating into pericytes or, at lesser extent, into endothelial cells (EC) [37], [56]–[58]. In contrast with these latter findings, a recent study demonstrated that MSCs inhibited capillary growth under certain conditions [59]. In particular, after addition of MSCs to EC-derived capillaries in matrigel at EC to MSCs ratio of 1∶1 or 1∶3, MSCs migrated toward the capillaries, established gap junctional communication (GJC) with ECs and secreted higher amounts of reactive oxygen species (ROS). This latter event culminated into EC apoptosis and capillary degeneration [59]. Furthermore, MSCs injected locally in an *in vivo* melanoma model, inhibited tumor growth by abrogating tumor angiogenesis [59]. These results, together with those we have reported in the *in vivo* s.c. NB model, indicate that the effects of MSCs on tumor angiogenesis may be heterogeneous possibly in relation to the microenvironment where they take place.

In conclusion, this study demonstrates that MSCs administered systemically did not home to NB tumors, under our experimental conditions, in either immunocompetent or immunodeficient pseudometastatic models. However, intratumoral injection of MSCs in established NB s.c. nodules inhibited tumor growth. Consistent with our results, Karnoub et al. demonstrated that MSCs exerted a pro-metastatic effect when co-injected with breast cancer cells but not when injected distantly from tumor cells. Accordingly, MSCs injected at a distant site were not detected in any pulmonary metastasis [28].

Our finding that systemically administered MSCs did not localize to NB tumors, under our conditions, nor influence their growth is not in support of the use of MSCs as vehicles for selective delivery of antitumor drugs at NB tumor site.

However, it is important to underline that the results obtained in our studies may be not generalized to all clinical scenario of NB patients but may be related to specific and unique features of the NB cell lines used.

In addition, the possibility exists that recently administered chemotherapy and/or radiotherapy create conditions in the tumor microenvironment, such as release of DAMPs and other chemoattractants, that allow MSC recruitment to the tumor site [21].

## Materials and Methods

### Mesenchymal stem cell isolation, characterization and culture

hMSCs were expanded *in vitro* from the bone marrow of healthy donors after informed written consent by G. Gaslini Institute Ethycal Committee (composed by Dr.ssa A. Comelli, Dr. S. Del Buono, Dr. G. Gavotti, Prof. A. Martini, Prof. L. Moretta, Prof. P. Moscatelli, Dr.ssa R. Rossi and Dr.ssa G. Sinaccio and supervised by Dr. S. Del Buono) on 09/02/2006. Mononuclear cells were isolated by gradient centrifugation at 2500 rpm for 30 minutes on Ficoll-Hystopaque-1077 (Sigma, St. Louis, MO; 1077 gr/ml density), washed twice with phosphate-buffered saline (PBS; Sigma), counted and plated at a concentration of 20–30×10^6^ cells/75-cm^2^ flask in Mesencult basal medium supplemented with Mesenchymal Stem Cell Stimulatory Supplement (StemCell Technologies, Vancouver, BC, Canada). After 1 week culture at 37°C in an atmosphere of 5% CO2, non adherent cells were removed, and the medium was replaced every other day. hMSCs were trypsinized when the cultures reached 80–100% confluence. The purity of hMSC suspensions was assessed by flow cytometry utilizing the following monoclonal antibodies: anti-CD34 fluorescein isothiocyanate (FITC) (BD Biosciences Pharmingen, Franklin Lake, NJ, USA), CD45 FITC (Caltag Laboratories, Burlingame, CA, USA), CD14 phycoerytrin (PE) (BD Biosciences Pharmingen), CD90 PE (BD Biosciences Pharmingen), CD73 PE (BD Biosciences Pharmingen), CD105 FITC (Diaclone, Besancon, France), CD44 FITC (BD Biosciences Pharmingen) and CD29 PE (BD Biosciences Pharmigen). As expected, the immunophenotype of hMSCs, after two-three passages in culture, was the following: CD34^−^ (0%), CD14^−^ (1,5%), CD45^−^ (0%), CD90^+^ (99%), CD105^+^ (96%), CD73^+^ (99%), CD44^+^ (98%), CD29^+^ (73%) cells (Fig. S2). The ability of MSCs to differentiate into the adipogenic and osteogenic lineageswas assessed as described [1]. In brief, MSCs were cultured in adipogenic medium (Cambrex, Walkersville, MD) for 3 weeks as well as in osteogenic medium (Cambrex) for 2 weeks. Histochemical analysis of cell layers was then performed by Oil Red Oil staining (Bioptica, Milan, Italy) for adipogenic differentiation and alizarin red dye staining for osteogenic differentiation (Sigma). hMSCs used for all the following experiments were cultured for no longer than 4 passages *in vitro*. mMSCs were isolated from bone marrow of 6- to 8-week-old A/J mice that was flushed out of tibias and femurs. After 2 washings by centrifugation at 1500 rpm (352 g) for 5 minutes in PBS, cells were plated in 75-cm^2^ tissue-culture flasks at the concentration of 0.3 to 0.4×10^6^ cells/cm^2^ using Murine Mesencult as medium (Stem Cell Technologies). Cells were kept in a humidified 5% CO2 incubator at 37°C, and the medium was refreshed every 3 to 4 days, for about 4 to 5 weeks; then, only adherent cells were collected following 10-minute incubation at 37°C with 0.05% trypsin (Sigma). After the first cut and for the subsequent 4 or 5 passages, the cells were plated in 25-cm^2^ flasks at 1.2 to 2.0×10^4^ cells/cm^2^. For the following passages, cells were routinely seeded at 4 to 10×10^3^ cells/cm2 reaching confluence after 4 to 5 days. The phenotype of mMSCs was routinely verified by flow cytometry using the following antibodies: CD11b FITC (Serotec (Oxford, United Kingdom), CD34 PE (Serotec) CD45 CyC (BD Biosciences Pharmingen), CD9 FITC (BD Biosciences Pharmingen) and Sca-1 PE (BD Biosciences Pharmingen).

mMSC and hMSC were infected with retroviral luciferase vector as follows. The retroviral luciferase vector was obtained by cloning the firefly luciferase gene (ffluc), excised from the pGL3 control vector (Promega), into the retroviral pLXIN bicistronic vector (Clontech, Saint-Germain-en-Laye France), to obtain pL-Luc-IN. Retroviruses were prepared by LipofectAMINE 2000 (Invitrogen, San Giuliano Milanese, Italy) transient transfection of pL-Luc-IN into the helper virus free Phoenix-Ampho packaging cells (gift from Nolan Lab, Stanford University, Stanford, CA). The retrovirus-containing supernatant was collected 48 h post-transfection and used to infect mMSC and hMSC cells. After 48 h, the transduced cells were selected in the presence of 0,5 mg/ml of G418 (Calbiochem, Rome, Italy). The resulting cells were denominated ffLuc-mMSCs and ffLuc-hMSCs, and presented phenotypic characteristics and *in vitro* growth properties virtually identical to the parental cells (data not shown).

### Tumor cell lines

SH-SY5Y [60], ACN [61], IMR-32 [62], GI-ME-N and GI-LI-N [63], [64], Htla-230 (kindly provided by Prof. Emil Bogenmann), human NB cell lines and NXS2 murine NB cell line [65] were cultured in DMEM medium (Euroclone, Milan, Italy) supplemented with L-glutamine, penicillin/streptomycin, nonessential amino acids and 10% fetal bovine serum (FBS) (Sigma) (complete medium). The conditioned medium was collected from NB cells cultured in 25-cm^2^ flasks for 2 days. The conditioned medium was sequentially centrifuged first at 3000 g and then at 15000 g for 10 minutes, filtered through 0.22-µm pore size filters (Costar, Cambridge, MA), and stored at −80°C.

### Proliferation assay

NB cell lines were plated in 96-well plates (Htla-230 6×10^3^; SH-SY5Y 4×10^3^, ACN 2×10^3^ cells per well) in complete medium in presence or absence of hMSCs at different ratio (1/1; 4/1; 10/1) for 72 hours. Before co-incubation, hMSCs were irradiated at 50 Gy. Cells were then incubated for 18 hours with 0.5 µCi (0.0185 MBq) ^3^H-thymidine (Amersham Bioscience, Little Chalfont, Buckinghamshire, UK). Cells were subsequently harvested onto glass fiber filters (Skatron Instruments, Tranby, Norway) by using a multiple automated sample harvester (Flow Laboratories, Milan, Italy), dried, and the incorporated ^3^H-thymidine was analyzed by using a liquid scintillation counter (Tri-Carb 4530; Packard Instruments Company, Downers Grove, IL).

### *In vitro* migration and invasion assay

Migration assay was performed in transwell dishes (BD Bioscience) 6.5 mm in diameter with 8 μm pore filters. hMSCs (2×10^4^) were plated in the upper wells while NB cells (5×10^4^) or NB cell supernatants (600 λ) were dispensed in the lower chamber. Negative and positive controls were represented by serum-free medium (DMEM) and PDGF-BB (R&D System Minneapolis, MN) at 100 ng/mL, respectively. After 24 hours incubation the upper side of the filter was washed with cold PBS, and cells remaining on the upper face of the filters were removed with a cotton wool swab. Invasion was quantified by counting migrated cells under the microscope after cell staining. Transwell filters were stained using May Grunwald-Giemsa (Sigma) staining and migrated cells were counted under the microscope.

Invasion assays were performed in Matrigel®-coated invasion chambers (BD Biosciences) 6.5 mm in diameter with 8 μm pore filters. SH-SY5Y, Htla-230, GI-LI-N NB cells incubated or not with hMSCs in a transwell system for 24 hours were placed in the upper wells on Matrigel®-coated invasion chambers (BD Biosciences) and separated by an 8 μm membrane from bone marrow sample isolated from an healthy donor after informed consent or CXCL12 (R&D Systems) at 100 ng/mL. For inhibition of CXCR4/CXCL12 axis, the antagonist of CXCR4 AMD3100 (Sigma Aldrich) was added into the lower chamber at 5 μM. After 24 hrs, NB cells that had invaded into the lower chamber were stained and counted after May Grunwald-Giemsa. CXCR4 surface expression was evaluated on NB cells incubated with CXCR4-FITC mAb as well as isotype-matched control mAbs (BD Bioscience) for 30 min at 4°C, and then analyzed by flow cytometry with a FACScan instrument (Beckmann Coulter Gallius Cytometry).

### *In Vivo* Therapeutic Studies in Mice

All experiments involving animals were reviewed and approved by the Ethical Committee for animal experimentation (CSEA) as Animal use project n. 236 communicated to the Italian Ministry of Health having regard to the article of the D.lgs 116/92.

#### Pseudometastatic tumor model in an immunodeficient host

Five-week-old female athymic (Nude-nu) mice were purchased from Harlan Laboratories (Harlan Italy, S.Pietro al Natisone, Italy) and housed under specific pathogen-free conditions. To evaluate when pseudometastatic NB tumors became established, mice were i.v. injected with ffLUC-Htla-230 tumor cells at a concentration of 3×10^6^/mouse. Animals were imaged at different times after tumor cell inoculum by IVIS™ Imaging System.

To examine the effect of hMSCs on metastatic tumor growth, athymic (Nude-nu) mice were i.v. injected with 3×10^6^ Htla-230 tumor cells. Mice were randomly assigned to receive i.v. hMSCs (1×10^6^ cells/mouse or 3×10^6^ cells/mouse) 2 weeks after tumor cell inoculum or saline solution (control mice). In some experiments, NB bearing mice as well as control mice were i.v. injected with ffLUC-hMSCs (1×10^6^ cells/mouse) and then imaged 1, 7, 14, 21 days after ffLUC-hMSC inoculum. Body weight and general physical status of the animals were recorded daily, and mice were killed by cervical dislocation after being anesthetized with xilezine (Xilor 2%, Bio98 Srl, Milan, Italy), when they showed signs of poor health, such as abdominal dilatation, dehydration, or paraplegia. Tumors were fixed in 10% buffered-formalin solution and embedded in paraffin. For morphological analysis, 4–μm-thick sections were cut from paraffin blocks and stained with hematoxylin-eosin (Sigma).

#### Orthotopic tumor model in an immunodeficient host

Five-week-old female athymic Nude mice were anesthetized with ketamine (Imalgene 1000, Merial Italia SpA., Milan, Italy), subjected to laparotomy, and injected with SH-SY5Y cells (1×10^6^ cells in 20 µL of saline solution) in the capsule of the left adrenal gland. Mice were randomly assigned to receive i.v. hMSCs (1×10^6^ cells/mouse). No mice died as a result of this treatment. The body weight and general physical status of the mice were recorded twice a week, and the mice were killed when tumors of the killed mice reached 1000–1200 mm^3^.

#### Subcutaneous tumor model in an immunodeficient host

Five-week-old female athymic (Nude-nu) were injected with human NB ACN cells (3×10^6^ cell/mouse) s.c. into the right flank. When the tumors reached the dimensions of 100 mm^3^, hMSCs (1×10^6^ cell/mouse) or saline solution (control group) were intratumorally injected. Tumor sizes were measured every day and the volume was calculated using the formula π/6 [w_1_ × (w_2_)^2^], where w_1_ represents the largest tumor diameter and w_2_ represents the smallest tumor diameter. The mice were killed when tumors reached 1500–2000 mm^3^.

#### Pseudometastatic tumor model in immunocompetent mice

To evaluate when pseudometastatic NB tumors became established, six-week.old female A/J mice (Harlan), were injected i.v. with murine syngeneic NB ffLUC-NXS2 cell line (2×10^5^ cells/mouse) and imaged at different times after tumor cell inoculum by IVIS™ Imaging System.

In a set of experiments, healthy and NB bearing animals were treated with intravenous injections of ffLuc-mMSCs (1×10^6^ cells/mouse) 7 days after NXS2 tumor cell inoculum. The animals were imaged 8, 15, 22, 29 days after tumor cell inoculum. Long-term survival was used as main criteria for the evaluation of *in vivo* effect of MSCs.

### *In vivo* bioluminescent imaging

*In vivo* bioluminescent imaging was performed in NB bearing and control immunocompetent and athimic mice with a ultra low-noise, high sensitivity cooled CCD camera, mounted on a light-tight imaging chamber (IVIS 100 System™, Xenogen, Roissy, France). Tracking, monitoring and quantification of signals were controlled by the acquisition and analysis software Living Image® (Xenogen). After cell inoculation, 150 mg/kg D-luciferin was administered i.p. (3 mg/mouse), and luminescence was captured from both dorsal and ventral views. D-luciferin was administered to anesthetized (1–3% isoflurane) animals 15 minutes (min) before image acquisition. Anesthetized mice were then placed in the IVIS™ Imaging System and imaged. Three-four mice were imaged at each time.

### Immunohistochemical and immunofluorescence analysis

Tissue samples were fixed in 20% buffered formalin, routinely processed and embedded in paraffin.

Briefly, 5 µm thick sections were cut from formalin fixed, paraffin embedded blocks, deparaffinized with xylene and rehydrated by passages through decreasing concentrations of ethanol (from 100% to 70%). Tissue were permeabilized with a solution of TritonX100 (0.1% in PBS) for 8 minutes at room temperature. Tissue slides were boiled for 15 min in 1 mM EDTA (pH 8.0) in a microwave oven. Endogenous peroxidase activity was blocked by a 10 min. incubation at room temperature with methanol containing 3% H_2_O_2_. The sections were then washed in PBS and incubated 1 hour at room temperature with Ki67 (1∶100, monoclonal mouse anti-human Clone MIB-1; Dako, Milan, Italy) and CD90 (1∶100, monoclonal rabbit anti-human Clone EPR3132; Abcam, Cambridge, UK) Ab. Isotype-matched Ab were as negative control to exclude non-specific reactivity. After the incubation with the primary Ab, tissue sections were rinsed twice in PBS, and incubated for 30 min at room temperature with Dako Envision System horse radish HRP Mouse and Rabbit for Ki67 and CD90/Thy1 respectively. Tissue sections were then washed in PBS, and peroxidase activity detected by a 6–10 min incubation at room temperature with Liquid diaminobenzidine (DAB) Substrate Chromogen System (Dako). Counterstaining was performed with Mayer's hematoxylin (Sigma). TUNEL assay on tumour sections was performed following manufacturers' instructions. Briefly, tissue sections were deparaffinised, permeabilized, and stained with a solution containing terminal deoxynucleotidyl transferase and nucleotide mixture for 1 hour at 37°C. After washing, the slides were dried at room temperature and counter-stained with DAPI (Vectashield mounting, Vector Laboratories).

For immunofluorescence analysis, tissue sections were incubated for 1 hour at room temperature with rabbit anti human active caspase-3 (1∶50; Abcam, Cambridge, UK). Isotype-matched Ab were used in all antibody-staining experiments to exclude non-specific reactivity. Binding of the primary antibodies was detected with Fluorescein goat anti-rabbit IgG (Invitrogen, Molecular Probe). After washing, the slides were counterstained with DAPI (Vectashield mounting, Vector Laboratories) and coverslipped. Digital images were acquired using a Nikon E-1000 fluorescence microscope (Nikon Instruments, Tokyo, Japan) equipped with appropriate filter sets and the Genikon imaging system software (Nikon Instruments).

### Statistical analysis

The statistical significance of differences between experimental and control groups was determined by Unpaired t test with Welch's correction using GraphPad Prism 3.0 software (GraphPad Software, Inc, El Camino Real, San Diego, CA). The statistical significance of differential survival between experimental groups of animals was determined by Kaplan-Meier curves and log-rank (Peto) test by the use of StatDirect statistical software (CamCode, Ashwell, UK).

All statistical tests were two-sided, and P values less than .05 were considered statistically significant.

## Supporting Information

Figure S1 **Survival curve of human neuroblastoma orthotopic bearing mice treated with human mesenchymal stem cells.** Athymic mice were injected with human NB SH-SY5Y cells (2×10^6^ cells/mouse) in the fat pad of the adrenal gland. Eight mice were treated with hMSCs (1×10^6^ cells/mouse) whereas seven control mice received saline solution. Survival curves were constructed by using the Kaplan–Meier method. Statistical analysis of different treatment groups was performed by Peto's log-rank test.
(TIF)

Figure S2 **Immunophenotype of human mesenchymal stem cells.** hMSC were stained with antibodies against surface specific markers (dark profile) or an isotype-matched mAb (open profile) and analyzed by flow cytometry. This experiment is representative of the three performed.
(TIF)
