# Supplementary figures and images for: Close Interactions between Mesenchymal Stem Cells and Neuroblastoma Cell Lines Lead to Tumor Growth Inhibition
Source: PLoS One. 2012 Oct 31;7(10):e48654. doi: 10.1371/journal.pone.0048654 (PMC3485378; doi:10.1371/journal.pone.0048654)

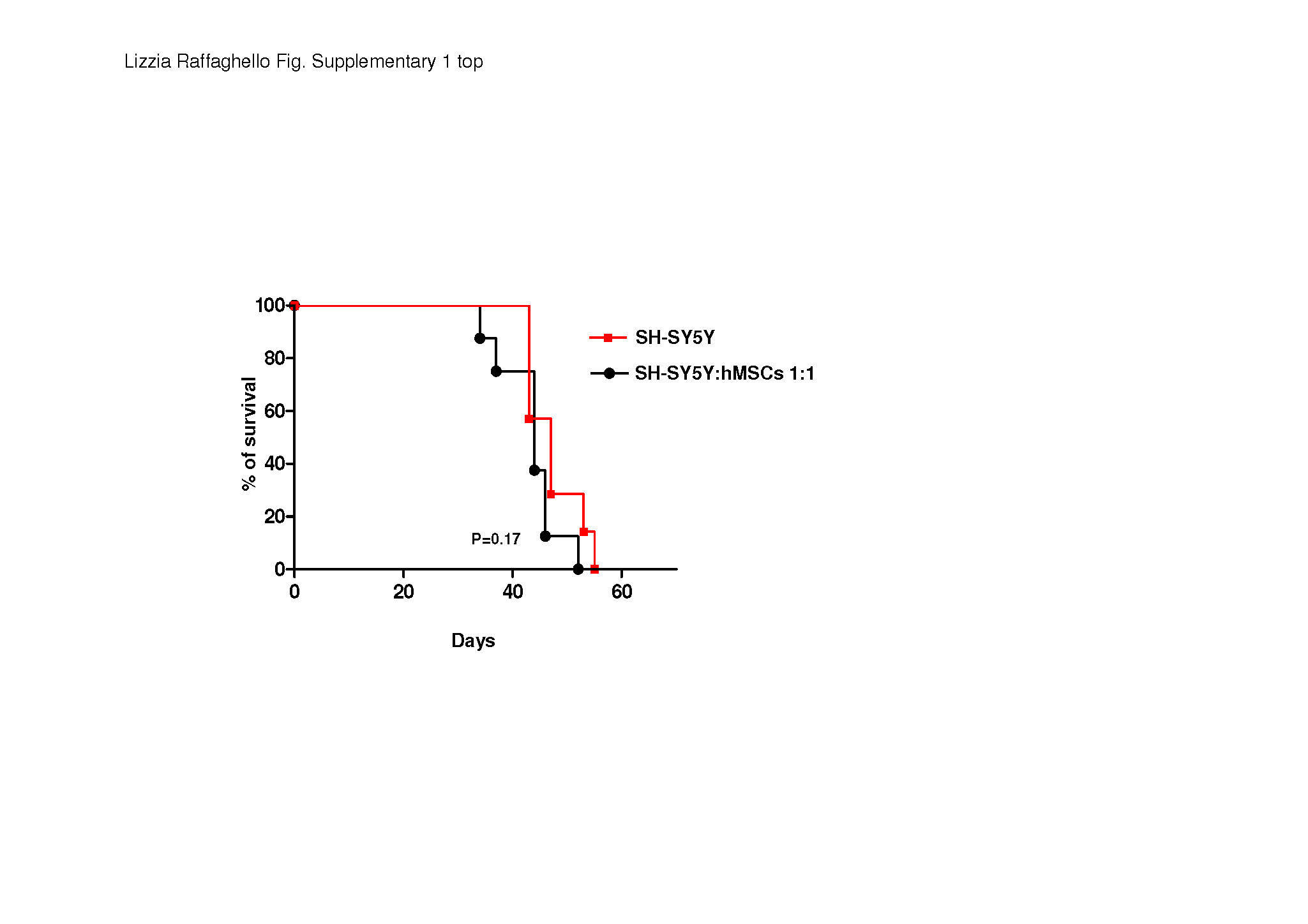

Supplement: Figure S1 — Survival curve of human neuroblastoma orthotopic bearing mice treated with human mesenchymal stem cells. Athymic mice were injected with human NB SH-SY5Y cells (2×10^6^ cells/mouse) in the fat pad of the adrenal gland. Eight mice were treated with hMSCs (1×10^6^ cells/mouse) whereas seven control mice received saline solution. Survival curves were constructed by using the Kaplan–Meier method. Statistical analysis of different treatment groups was performed by Peto's log-rank test. (TIF) [file pone.0048654.s001.tif]

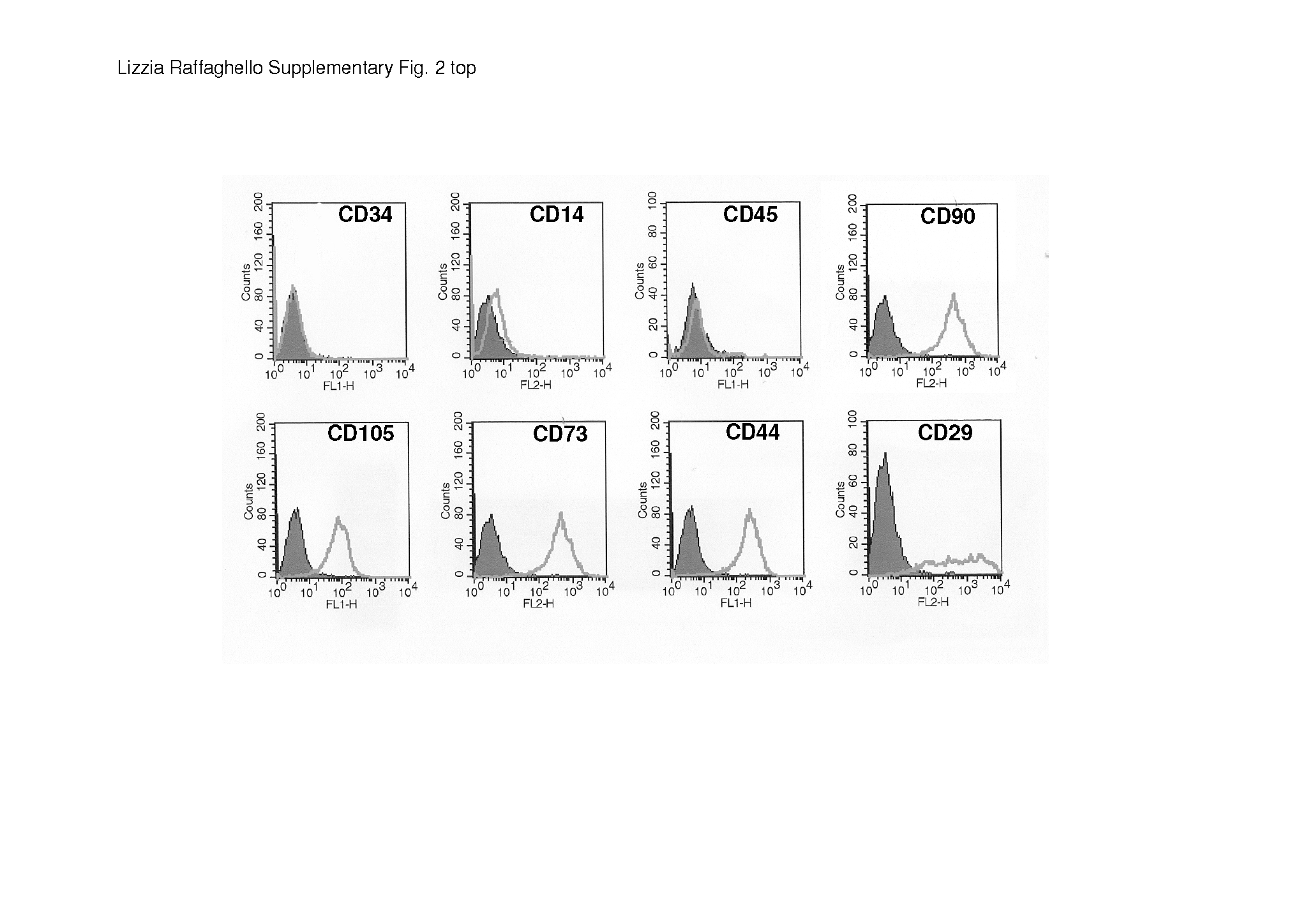

Supplement: Figure S2 — Immunophenotype of human mesenchymal stem cells. hMSC were stained with antibodies against surface specific markers (dark profile) or an isotype-matched mAb (open profile) and analyzed by flow cytometry. This experiment is representative of the three performed. (TIF) [file pone.0048654.s002.tif]
